# Comparison of the Potential Ecological and Human Health Risks of Heavy Metals from Sewage Sludge and Livestock Manure for Agricultural Use

**DOI:** 10.3390/toxics9070145

**Published:** 2021-06-24

**Authors:** Baoling Duan, Qiang Feng

**Affiliations:** College of Resources and Environment, Shanxi University of Finance and Economics, Taiyuan 030006, China; sxcddbl@sxufe.edu.cn

**Keywords:** organic waste, pollution index, health risk index, risk heavy metals, the Yanmenguan Cattle Herbivorous Livestock Area

## Abstract

Sewage sludge and livestock (chicken, swine and cattle) manure samples were collected from the Yanmenguan Cattle Herbivorous Livestock Area to compare the potential ecological and human health risks caused by heavy metals contained in them. In this study, the Class II level of Quality Control of Imported Organic Fertilizers is selected as the limit standard value of heavy metals. Based on the mean content values, no heavy metal in cattle manure was higher than the limit standard value; the content of Cu in swine manure was higher than the limit of Cu; the content of Zn in sewage sludge, chicken manure and swine manure were all higher than the limit of Zn; and the content of Cr in sewage sludge and chicken manure were all higher than the limit of Cr. Results indicated that sewage sludge and livestock manure all had high contents of Zn, Cu and Cr. The mean pollution index (PI) suggested that Cu, Zn, As and Cr in sewage sludge and livestock manures all induced potential ecological risks. According to the mean Nemerow’s synthetic pollution index (PN) values, swine manure had the highest potential ecological risk for agricultural use. Daily exposure to Cu, Zn and Cr was higher than other heavy metals from sewage sludge and livestock manures, and heavy metal exposure was always higher for children than adults, with ingestion as the main pathway. Non-carcinogenic risk was caused mainly by Cu and Cr, based on the higher hazard quotient (HQ) values for adults and children. There was no non-carcinogenic risk for all people, except exposure of Cu from swine manure for children, which was 1.76 times higher than the threshold value of 1. According to the mean hazard index (HI) values, only swine manure had a non-carcinogenic risk for children. As the carcinogenic risk index (Risk) values were continuously greater for As than Cd, As had a higher carcinogenic risk than Cd. There was no carcinogenic risk for any single heavy metal, although As exposure from sewage sludge was found to have an inapparent carcinogenic risk for both adults and children. Regarding the RISK value, sewage sludge had an unacceptable carcinogenic risk for adults and children, and swine manure had an unacceptable risk for children only. In general, for both non-carcinogenic and carcinogenic risks, ingestion was the main pathway, and children were more sensitive than adults. Comparing the four kinds of organic waste, cattle manure was the safest for agricultural use in terms of ecological and human health risks. In multiple comparisons, swine manure was significantly different regarding potential ecological risk and non-carcinogenic risk, and sewage sludge was significantly different regarding carcinogenic risk.

## 1. Introduction

Sewage sludge and livestock manure are rich in nutrients, such as organic matter, nitrogen, phosphorus, and potassium, which are necessary for plant growth and improved crop yields [1,2]. Hence, sewage sludge and livestock manure are widely used in agriculture around the world and are recommended as land fertilizer by the government of China [3,4]. In Shanxi, 42.66% of sewage sludge is disposed of by land use as soil conditioners or fertilizers, and 87.8% of livestock manure is directly returned to nearby fields [4,5].

The way sewage sludge and livestock manure are used as soil conditioners is regarded as a method of waste recycling, and it is the most economical means for handling this waste [1,6,7,8]. However, in addition to the advantages of the nutritious substances contained in sewage sludge and livestock manure that can increase plant yield and improve soil properties, the toxic materials they contain, especially heavy metals, also enter into the soil [6,8,9,10]. In the process of wastewater treatment, 50–80% of heavy metals are transferred into sewage sludge [11]. In addition, most heavy metals are excreted in livestock manure; large quantities of them are added to fodder to prevent diseases and improve the growth of livestock, but their usage is low [12,13]. Due to its non-biodegradability, persistence and toxicity, the agricultural use of sewage sludge and livestock manure causes heavy metal pollution of the soil environment [14,15,16,17]. Furthermore, heavy metals in sewage sludge and livestock manure will not only enter into the soil, but also other ecosystems, such as the atmosphere, groundwater, surface water and biosphere, as all ecosystems are mutually connected [1,18,19,20]. Therefore, using sewage sludge and livestock manure as a soil fertilizer will not only potentially cause ecological risk to the ecosystem, but also pose a threat to human health through groundwater, the human food chain and other ecosystems [16,18,19,21]. Heavy metal has become a critical factor in the agricultural use of sewage sludge and livestock manure [22].

In order to prevent the adverse effects of the agricultural use of sewage sludge and livestock manure induced by heavy metals, potential ecological and human health risks must be assessed [23,24]. The assessment of the potential ecological risks of heavy metals commonly uses the geo-accumulation index, single-factor pollution index, Nemerow’s synthetic pollution index, and ecological risk index [25,26,27,28]. Most of the assessments of the human health risks of heavy metals basically adopt the exposure models formulated by the United Stated Environmental Protection Agency (US EPA). However, there are fewer studies comparing the potential ecological and human health risks between sewage sludge and livestock manure. In addition, human health risk assessment often focuses on adults, but children should receive special attention in health risk assessment due to their low tolerance to toxins and inadvertent behavior like ingestion, putting them in contact with significant quantities of sewage sludge and livestock manure. In this study, in order to assess the differences between sewage sludge and livestock manure in terms of the risks they pose to environment and human health, a comparison was performed.

The aims of this present study were as follows: (a) to identify the concentration of heavy metals in sewage sludge and livestock manure sampled from the Yanmenguan Cattle Herbivorous Livestock Area in the north of Shanxi; (b) to assess the potential ecological risks of heavy metals in sewage sludge and livestock manure and compare the differences; (c) to evaluate heavy metal exposure from sewage sludge and livestock manure and distinguish the difference in exposure of adults and children; and (d) to identify and compare non-carcinogenic and carcinogenic risk between sewage and livestock manure for adults and children.

## 2. Materials and Methods

### 2.1. Sampling and Chemical Analysis

Sewage sludge and livestock manure (chicken, swine, and cattle) were sampled from the Yanmenguan Cattle Herbivorous Livestock Area, north of Shanxi, China. Sewage sludge was collected from five municipal wastewater treatment plants with the largest production in the study area; for chicken, swine, and cattle manure, five, six, and seven samples, respectively, were collected from intensive livestock farms. To enhance the sample’s representativeness, four subsamples were collected from four different sites in the storage pile at each wastewater treatment plant and four different sites in the manure storage area at each farm. Then, the four subsamples were blended together as one sample.

At room temperature, samples were air-dried in a clean environment, sieved through a mesh with a pore size of 0.14 mm, and then placed in brown glass bottles. Samples were weighed and digested with HNO_3_ using a microwave digestion system based on US EPA Method 3051B [29]. Cu, Zn, Pb, and Cr were analyzed using an atomic absorption spectrophotometer; Cd was analyzed using a graphite furnace atomic absorption spectrophotometer; and As was analyzed using an atomic fluorescence spectrometer. The Chinese national standards GB/T 15555.2-1995, GB/T 15555.2-1995, GB/T 15555.2-1995, GB/T 15555.6-1996, GB/T 17141-1997, and GB/T 22105.2-2008 were used to perform chemical analysis, and solutions used for the calibration of the instruments were implemented in these standards. Certified reference material was also used to control the quality. Accuracy, precision and recovery were checked by testing the certified reference material. The method detection limit was confirmed by testing blind samples 11 times. When each batch of samples was tested, two blank samples and reference samples were detected at the same time. Triplicate samples were determined, and then the mean value of the result was the final concentration of heavy metals. Results are shown in Table 1.

### 2.2. Ecological Risk Assessment

The single-factor pollution index (PI) was developed and used to assess the contamination of a single heavy metal, and it was evaluated to express the pollution level by comparing with standard values. It is defined as the following equation [30,31]:

PI = C_i_/S_i_

where PI is the single-factor pollution index of the ith heavy metal, C_i_ is the concentration of the ith heavy metal (mg/kg) in sewage sludge and livestock manure, and S_i_ is the limit standard value of the ith heavy metal (mg/kg). In this paper, S_i_ is represented by the Class II of Quality Control of Imported Organic Fertilizers; the corresponding standard values (S_i_) for Cu, Zn, Cd, Pb, As and Cr are 300, 300, 1.0, 100, 10 and 100 mg/kg, respectively [32]. The contamination level of heavy metals is classified into five grades based on the PI value: no contamination: PI ≤ 1.0; low contamination: 1.0 < PI ≤ 2.0; moderate contamination: 2.0 < PI ≤ 3.0; strong contamination: 3.0 < PI ≤ 5.0; and very strong contamination: PI > 5.0 [30,31].

To assess the synthetic pollution of all heavy metals, Nemerow’s synthetic pollution index (PN) was used, and the equation is as follows [33]:

$$\mathrm{PN}=\sqrt{\frac{P_{i,\mathrm{ave}}^{2}+P_{i,\max}^{2}}{2}}$$

where PN is the synthetic pollution index, $P_{i,\mathrm{ave}}$ is the average value of the single-factor pollution index of the ith heavy metal, and $P_{i,\max}$ is the maximum value of the single-factor pollution index of the ith heavy metal. According to the PN value, the pollution level can be divided into 5 classes: safe: PN ≤ 0.7; warning line of pollution: 0.7 < PN ≤ 1.0; slight pollution: 1.0 < PN ≤ 2.0; moderate pollution: 2.0 < PN ≤ 3.0; and heavy pollution: PN > 3.0 [31,34].

PI and PN focus on different aspects of toxic element pollution: PI expresses the pollution situation of one single heavy metal, and PN emphasizes contamination caused by the total of heavy metals in the environment, and it synthesizes not only their average level, but also their maximum level [35].

### 2.3. Human Health Risk Assessment

Human health risk assessment involves the interaction of environmental pollutants and human health. It can be considered as a quantitative description of the risk that environmental pollutants will cause harm to human health [36]. Based on the US EPA Part 503 rule, heavy metal exposure in humans has two pathways, ingestion and inhalation [37]. According to the International Agency for Research on Cancer (IARC) and the World Health Organization (WHO), Cd and As are classified as carcinogenic pollutants [38,39].

#### 2.3.1. Exposure Assessment

Human exposure to heavy metals is expressed as an average daily dose (ADD), based on the health risk model recommended by the US EPA, determined by the following equations [40,41,42,43]:

$$\mathrm{ADD}={\mathrm{ADD}}_{\mathrm{ingest}}+{\mathrm{ADD}}_{\mathrm{inhale}}\;{\mathrm{ADD}}_{\mathrm{ingest}}=\frac{C\times {\mathrm{IR}}_{\mathrm{ingest}}\times \mathrm{EF}\times \mathrm{ED}}{\mathrm{BW}\times \mathrm{AT}}\times \mathrm{CF}\;{\mathrm{ADD}}_{\mathrm{inhale}}=\frac{C\times \mathrm{InhR}\times \mathrm{EF}\times \mathrm{ED}}{\mathrm{PEF}\times \mathrm{BW}\times \mathrm{AT}}$$

where ADD_ingest_ is the average daily dose for ingestion (mg·kg^−1^·day^−1^); ADD_inhale_ is the average daily dose for inhalation (mg·kg^−1^·day^−1^); ADD is the average daily total exposure dose (mg·kg^−1^); C is the concentration of heavy metals in sewage sludge and livestock manure (mg·kg^−1^); IR_ingest_ is the ingestion rate of heavy metals, which is 100 mg·day^−1^ for adults and 200 mg·day^−1^ for children [44]; EF is the exposure frequency, with 350 days·year^−1^ [44]; ED is the exposure duration, which is 30 years for adults and 6 years for children; BW is average body weight, which is 70 kg for adults and 16 kg for children [37]; AT is the average time, and for non-carcinogens is equal to ED × 365 days and for carcinogens is equal to 70 years (lifetime) × 365 days [44]; CF is a conversion factor (1 × 10^−6^); InhR is the inhalation rate [44], and is 7.6 m^3^·day^−1^ for children and 20 m^3^·day^−1^ for adults [34]; and PEF is the particle emission factor (1.36 × 109 m^3^·kg^−1^) [43].

#### 2.3.2. Non-Carcinogenic Risk Assessment

The hazard quotient (HQ) was applied to estimate the non-carcinogenic risk of a single heavy metal, using the following equation [40,45]:

$${\mathrm{HQ}}_{\mathrm{ij}}=\frac{{\mathrm{ADD}}_{\mathrm{ij}}}{{\mathrm{RfD}}_{\mathrm{ij}}}$$

where HQ_ij_ is the hazard quotient of the ith heavy metal via the jth pathway; ADD_ij_ is the average daily dose for the ith heavy metal via the jth pathway (mg·kg^−1^·day^−1^); and RfD_ij_ is the risk reference dose of the ith heavy metal via the jth pathway (mg·kg^−1^·day^−1^). The RfD values of Cu, Zn, Pb and Cr via ingestion and inhalation are the same: 0.004, 0.300, 0.038 and 0.005 mg·kg^−1^·day^−1^, respectively [39].

In order to assess the total non-carcinogenic risk of human exposure to different heavy metals through different pathways, the hazard index (HI) is introduced, using the following equation [46]:

$$\mathrm{HI}=\overset{n}{\underset{i=1}{\sum }}\overset{m}{\underset{j=1}{\sum }}{\mathrm{HQ}}_{\mathrm{ij}}$$

If HQ < 1 or HI < 1, there is no significant non-carcinogenic risk, which can be ignored; if HQ > 1 or HI > 1, there is significant non-carcinogenic risk, which will increase with increasing values of HQ or HI [40,41,42].

#### 2.3.3. Carcinogenic Risk Assessment

For individuals who are exposed to potential carcinogenic pollutants, the possibility of developing cancer in their lifetime is considered carcinogenic risk. It is represented by the carcinogenic risk index (Risk) with the following equation [38,40,41]:

$${\mathrm{RISK}}_{\mathrm{ij}}={\mathrm{ADD}}_{\mathrm{ij}}\times \mathrm{SF}$$

where Risk_ij_ is the carcinogenic risk index of the ith heavy metal via the jth pathway and SF is the carcinogenic slope factor (kg·day·mg^−1^). The SF values of As and Cd via ingestion and inhalation are the same: 1.5 and 6.1 kg·day·mg^−1^, respectively [39].

In order to assess the total carcinogenic risk of human exposure to As and Cd, the sum of the risk of all heavy metals is expressed as RISK and the equation is as follows [46]:

$$\mathrm{RISK}=\overset{n}{\underset{i=1}{\sum }}\overset{m}{\underset{j=1}{\sum }}{\mathrm{Risk}}_{\mathrm{ij}}$$

If Risk < 1 × 10^−6^, there is no carcinogenic risk; if 1 × 10^−6^ < Risk < 1 × 10^−4^, there is an inapparent carcinogenic risk, and the risk is acceptable; if Risk > 1 × 10^−4^, there is significant carcinogenic risk, and the risk is unacceptable. For total heavy metals via the two pathways, if RISK < 1 × 10^−5^, the carcinogenic risk is acceptable, and if RISK > 1 × 10^−5^, the carcinogenic risk is unacceptable [39,40,46]. Then, we applied one-way analysis of variance (one-way ANOVA) and the Duncan method for multiple comparisons to analyze different significance of heavy metal indices from different sources.

## 3. Results

### 3.1. Heavy Metal Concentration in Sewage Sludge and Livestock Manure

The heavy metal content in sewage sludge and different kinds of livestock manure was determined and is presented in Table 2. According to the mean values of concentration, heavy metals were ranked in decreasing order as follows: Zn > Cr > Cu > Pb > As > Cd for sewage sludge and chicken manure; Zn > Cu > Cr > As > Pb > Cd for swine manure; and Zn > Cu > Cr > Pb > As > Cd for cattle manure. Not only in sewage sludge but also in livestock manures, Zn, Cu, and Cr contents were higher.

The Cd, Pb, As and Cr content in sewage sludge is much higher than that in livestock manure, except for Cr in chicken manure, which is almost equal to that in sewage sludge. Furthermore, multiple comparisons indicated that sewage sludge was significantly different from livestock manure regarding the content of Cd, Pb, As and Cr, but not significantly different from chicken manure regarding the content of Cr. The highest Cu and Zn contents were found in swine manure, the second highest in chicken manure and the lowest in cattle manure. In the multiple comparisons, swine manure was significantly different from other organic waste regarding the content of Cu and Zn. This is due to the fact that in the breeding process, large amounts of Cu and Zn are added to the fodder, but are often less used by animals and not easy to decompose, so they are excreted in the manure [47]. Cattle manure contains the lowest amount of Cu and Zn, which may be due to the fact that cattle are mainly fed with silage and grass [48,49].

As shown in Table 3, the heavy metal limits formulated by different countries for agricultural use of sewage sludge are generally the same, except Canada has more rigid standards than other countries [50]. Around the world, although there are no special limits on heavy metals for organic waste, some European countries, such as Belgium, the Netherlands and Germany, have strict limits on heavy metals in compost [51]. In China, the standards for heavy metals in organic fertilizers, such as Control Standards for Urban Wastes for Agricultural Use (GB8172-87), Technical Specification for Animal Manure Composting (NY/T 3442-2019), and Organic Fertilizer (NY525-2012), were all lack of Cu and Zn indices. Compared with the compost standard, the heavy metal content limits are more relaxed for the agricultural use of sewage sludge. Hence, the Class II level of Quality Control Standards of Imported Organic Fertilizers was adopted in this study.

Compared with the threshold values, the contents of Cd and Pb in all samples were lower. The mean contents of Zn, As and Cr for sewage sludge, Zn and Cr for chicken manure, Cu and Zn for swine manure and no heavy metals for cattle manure were higher than the limit values.

### 3.2. Potential Ecological Risk Assessment

The assessment of potential ecological risks of sewage sludge and livestock manure are shown in Table 4. Zn, As and Cr in sewage sludge, Zn and Cr in chicken manure and Cu in swine manure all had low levels of contamination, based on PI values higher than 1; Zn in swine manure had a higher level of contamination according to its mean PI value being higher than 3. The results show that the potential ecological risk of sewage sludge and livestock manure mainly involved Zn and Cr. Multiple comparisons indicated that the potential ecological risk caused by swine manure was significantly different from other organic waste regarding Cu and Zn; the ecological risk induced by sewage sludge was significantly different regarding Cd, Pb and As; regarding Cr, sewage sludge and chicken manure showed no significant difference, and swine manure and cattle manure were the same, but they showed significant differences between them. Zn and Cr pollution in sewage sludge was caused by industrial production, and in livestock manure by feed supplementation and the animal’s metabolic characteristics (low usage and high excretion of heavy metals) [12,49]. Based on the mean PN values, the pollution severity ranking of sewage sludge and livestock manure was swine manure > chicken manure > sewage sludge > cattle manure. In the multiple comparisons, swine manure was significantly different from other organic waste; chicken manure and sewage sludge had no significant difference. In addition, the mean PN value for swine manure was higher than three, indicating that pig manure had heavy pollution for agricultural use; the mean PN values for sewage sludge and chicken manure were higher than two, showing that these had moderate pollution; and for cattle manure, the value was lower than 0.7, showing that it was safe for land use. These results suggested that sewage sludge, chicken manure and swine manure all had dangerous levels of heavy metals and should undergo more scrutiny for agricultural use.

### 3.3. Health Risk Assessment

#### 3.3.1. Exposure Assessment

Daily exposure to heavy metals in sewage sludge and livestock manure was quantified based on the method recommended by the US EPA. The exposure rates for adults and children were compared, as shown in Table 5.

The trends of heavy metal exposure were the same for adults and children and can be ranked in decreasing order based on the mean values of ADD: Zn > Cr > Cu > Pb > As > Cd for sewage sludge and chicken manure, and Zn > Cu > Cr > Pb > As > Cd for swine manure and cattle manure. Furthermore, the exposure trends of different kinds of heavy metals were the same for both adults and children. For Cu and Zn, exposure can be ranked as swine manure > chicken manure > sewage sludge > cattle manure; for As and Cd, sewage sludge > swine manure > chicken manure > cattle manure; for Pb, sewage sludge > cattle manure > chicken manure > swine manure; and for Cr, sewage sludge > chicken manure > cattle manure > swine manure. In sum, the magnitude of heavy metal exposure for both adults and children was consistent with the heavy metal content, but the exposure rates were higher for children than for adults. Differences between four kinds of organic wastes were the same as the status based on heavy metal contents according to the multiple comparisons.

#### 3.3.2. Non-Carcinogenic Risk Assessment

The non-carcinogenic risk for adults and children based on heavy metal exposure in different kinds of organic waste is shown in Table 6. The ranking of heavy metals according to mean HQ values for adults and children was the same; they can be ranked in decreasing order as Cr > Cu > Zn > Pb for sewage sludge and chicken manure and Cu > Cr > Zn > Pb for swine and cattle manure. Multiple comparisons indicated that the non-carcinogenic risk caused by Cu and Zn exposure from swine manure was significantly different to that for other organic waste; the non-carcinogenic risk caused by Pb and Cr from sewage sludge was significantly different to that for other organic waste. Due to their high content and low RfD, Cr and Cu had high non-carcinogenic risks for adults and children. Zn, which had the highest content in all organic wastes, had a low non-carcinogenic risk as its RfD value was the highest. Pb, which had the lowest content in all organic wastes and a large RfD value was shown to have the lowest non-carcinogenic risk among the four non-carcinogenic heavy metals.

For each type of organic waste, the contribution of heavy metals to non-carcinogenic risk for adults and children was the same. Based on mean HQ values, Cu, Zn, Pb and Cr accounted for 33.38%, 2.23%, 0.93% and 63.46% of the HI value for sewage sludge; 39.87%, 2.46%, 0.31% and 57.36% for chicken manure; 95.07%, 2.01%, 0.10% and 2.83% for swine manure; and 64.54%, 2.50%, 2.26% and 30.70% for cattle manure. It can be seen from the results that Cu and Cr were the heavy metals that showed the highest correlation with non-carcinogenic risk. Although the Zn content in all four organic wastes was high, it had the lowest non-carcinogenic risk.

The values of HQ_ingest_ and HQ_inhale_ were less than one for all heavy metals; however, the HQ_ingest_ value of Cu for children was 1.76. This indicated no non-carcinogenic risk for adults and children exposed to heavy metals in sewage sludge and livestock manure via either ingestion or inhalation, but a non-carcinogenic risk for children exposed to Cu from swine manure via ingestion. The differential values of HQ_ingest_ and HQ_inhale_ showed that ingestion was the major pathway of heavy metal exposure from sewage sludge and livestock manure for adults and children, and inhalation had an almost negligible contribution.

Regarding the mean value, calculated HQ values were all less than one for adults and children for sewage sludge and all kinds of livestock manure, except for children exposed to swine manure. This indicates no non-carcinogenic risk for adults or children exposed to all organic wastes, except children exposed to swine manure. Furthermore, the HQ values were higher for children exposed to sewage sludge and livestock manure than adults, with the same heavy metal content.

As shown in Table 6, the HI values for adults and children were 7.79 × 10^−2^ and 6.82 × 10^−1^ for sewage sludge, 7.34 × 10^−2^ and 6.42 × 10^−1^ for chicken manure, 2.12 × 10^−1^ and 1.85 for swine manure, and 2.18 × 10^−2^ and 1.91 × 10^−1^ for cattle manure. For both adults and children, the HI values for swine manure were the highest and for cattle manure were the lowest. In the multiple comparisons, swine manure is significantly different from other organic wastes regarding non-carcinogenic risk. Furthermore, only the agricultural use of swine manure had a non-carcinogenic risk for children.

#### 3.3.3. Carcinogenic Risk Assessment

The carcinogenic risk for adults and children exposed to agricultural sewage sludge and livestock manure was calculated, and results are shown in Table 7. The Risk values were higher for As than Cd for both adults and children based on the mean value, and they are all less than 1 × 10^−4^. This indicated that the carcinogenic risk was higher for As than Cd, and exposure to both heavy metals from sewage sludge and livestock manure did not generate a significant unacceptable carcinogenic risk for either adults or children. Furthermore, the Risk values for sewage sludge were between 1 × 10^−4^ and 1 × 10^−6^, suggesting that there was insignificant carcinogenic risk from the agricultural use of sewage sludge. Comparing the two pathways of As and Cd exposure for adults and children, ingestion was found to be the main source of carcinogenic risk based on higher Risk_ingest_ than Risk_inhale_ values [42]. In the multiple comparisons, the carcinogenic risk caused by As and Cd exposure from sewage sludge showed a significant difference from other organic wastes.

Furthermore, the mean RISK values, which represented the total carcinogenic risk of As and Cd, were less than 1 × 10^−5^, except that for sewage sludge for adults and children and swine manure for children. This indicated that the agricultural use of chicken and cattle manure does not pose a carcinogenic risk to adults and children, but the use of sewage sludge would pose a carcinogenic threat to both adults and children and the use of swine manure would pose a carcinogenic risk to children only. Based on the mean RISK values of the total carcinogenic risk, the agricultural use of cattle and chicken manure was safer than the use of sewage sludge and swine manure. The multiple comparisons indicated that sewage sludge was significantly different from other organic waste regarding carcinogenic risk.

## 4. Discussion

### 4.1. Heavy Metals in Sewage Sludge and Livestock Manure

The source of heavy metals in sewage sludge is anthropogenic activities [52]. Coking, mining, metallurgy and leather tanning located in the study area caused the Zn, Cr, Cu, and Pb pollution of sewage sludge. Low level pollution of As and Cd in sewage sludge may be caused by households, such as by the use of detergents [35]. China’s third-largest mining industry, located in the study area, would cause the content of Cu and Zn in sewage sludge to be significantly higher.

In raising livestock, heavy metals are widely added to fodder as feed additives to promote livestock growth and prevent disease [53]. Because the usage rate of heavy metals by livestock is low, they are mostly metabolized in the manure [21]. Cu is important for normal growth, Zn is essential for breeding and immunity [54], and Cr can improve growth performance and feed consumption and efficiency as well as can improve carcass quality and enhance body immunity [13,55]. Due to the vital function of Cu, Zn and Cr for livestock raising, the content of these in livestock manure is greater now than in the 1990s [53]. In order to promote the growth of swine, more Cu and Zn are added to their feed, so the contents of Cu and Zn in swine manure are higher than those in other livestock manure. The highest content of Cr appears in chicken manure, due to the fact that Cr can improve the laying rate of chickens, prolong the peak period of laying, increase the egg weight, improve the egg quality and reduce the amounts of broken eggs [13].

In order to pursue the growth rate of livestock, enhance disease resistance and control animal physiological metabolism, many farms add excessive amounts of heavy metals, especially in Cu, Zn and Cr. This leads to contents of Cu, Zn and Cr in chicken manure, swine manure and cattle manure that are greatly different in each farm [56]. Unlike swine and chickens, cattle are mainly fed with silage and grass; thus, the pollution of silage and grass would have a great influence on cattle manure [48,49].

### 4.2. The Potential Ecological Risk

Because of the complexity of sewage sludge and livestock manure, PI cannot reflect the potential ecological risk better than PN, which combines the pollution levels of all metals to accurately reveal the pollution levels of sewage sludge and livestock manure [35,57,58].

The pollution of sewage sludge was caused mainly by Zn, As, and Cr from nearby industries, such as mining, coking, metallurgy and leather tanning [4,35,52,58]. The pollution of chicken manure was caused mainly by Zn and Cr, which are added to fodder to improve immunity and laying rate [59,60]. The pollution of swine manure was caused mainly by Cu and Zn, which were added to feed to strengthen immunity and the metabolic rate, which is low for swine [61,62]. Cattle manure was the safest of the four organic wastes for agricultural use [49]. As found in other studies, heavy metal pollution of swine manure is higher than other livestock manure [56].

### 4.3. The Human Health Risk

Based on the same concentration, heavy metal exposure was higher for children than for adults; the adverse effects of heavy metal exposure in sewage sludge and livestock manure were more serious for children [38,63]. This may be due to the fact that children have a lower body weight than adults and engage in more outdoor activity than adults [39,63]. Comparing the two pathways of heavy metal exposure, the effect of inhaling was negligible, and ingesting was more prominent. This is in agreement with other studies [39,46,63].

The non-carcinogenic risk for agricultural use of swine manure is greater than other organic wastes, and children have a higher non-carcinogenic risk from the agricultural use of swine manure. Compared with adults, children should receive more attention when they are exposed to the same contamination [42]. This suggests that children’s capacity to deal with toxins is weaker than adults’ when the toxin content is the same [64,65].

Regarding carcinogenic risk, As is more effective than Cd [4]. Agricultural use of sewage sludge poses carcinogenic risk to both adults and children, and the use of swine manure poses carcinogenic risk to children only. This suggests that the RISK value is always higher for children than for adults and that children are more vulnerable to carcinogenic risk than adults if they are in the same polluted environment [64].

## 5. Conclusions

To compare the ecological and human health risks of using sewage sludge and livestock manure for agricultural use, samples were collected, and the heavy metals contained in them were determined. The contents of Zn, Cu and Cr were high in sewage sludge and livestock manure; the highest content of Cu and Zn was in swine manure, and the lowest in cattle manure. Compared with the secondary Quality Control Standards of Imported Organic Fertilizers, the mean values of Cu in swine manure, Zn in sewage sludge, chicken manure, and swine manure, Cd and Pb in none of them, As in sewage sludge, and Cr in sewage sludge and chicken manure were higher than the threshold values.

Based on the mean PI values, Cu, Zn, As and Cr in sewage sludge and livestock manures all induced an ecological risk, and Zn in swine manure had a high contamination level. Based on mean PN values, organic waste can be ranked in the following order: swine manure > chicken manure > sewage sludge > cattle manure.

The daily exposure of Cu, Zn and Cr from sewage sludge and livestock manure was higher for both adults and children. Swine exposed the highest Cu and Zn and sewage sludge exposed the highest Cr. Comparatively, heavy metal exposure for children was higher than adults, and ingestion was the main pathway. Based on the mean HQ values, Cu and Cr were the main heavy metals, causing non-carcinogenic risks for both adults and children. Based on the mean HI value, only children had a non-carcinogenic risk posed by swine manure, mainly via ingestion. For carcinogenic risk, As posed a higher risk than Cd for all organic waste. Combining the two carcinogenic heavy metals, sewage sludge for adults and children and swine manure for children present unacceptable carcinogenic risks based on RISK values higher than 1 × 10^−5^. For both non-carcinogenic and carcinogenic risk, ingestion is the main pathway of heavy metal exposure for adults and children, and children are more sensitive than adults to the same pollutant exposure.

Comparing sewage sludge and livestock manure, cattle manure is the safest organic waste for agricultural use in terms of ecological and human health risk. Due to children’s behavioral characteristics, they are more sensitive when they are in the same adverse environment as adults, so children should receive more attention to avoid the harmful effects of pollutants. Multiple comparisons suggested that swine manure was significantly different regarding potential ecological risk and non-carcinogenic risk, and sewage sludge was significantly different regarding carcinogenic risk.

## Figures and Tables

**Table 1:** Analytical accuracy, precision, recovery and method detection limit.

Heavy Metal	Confidence Interval (mg/kg)	Certified Value (mg/kg)	Measured Value (mg/kg)	Accuracy (%)	Precision (%)	Recovery (%)	Method Detection Limit (mg/kg)
Cu	433–531	482	470.96	3.68	−2.29	94.61	0.907
Zn	1060–1420	1240	1229.59	5.02	−0.84	94.51	0.984
Cd	56.9–64.2	60	63.01	1.43	5.01	105.92	0.0063
Pb	143–165	154	147.79	3.38	−4.03	92.77	0.318
As	202–256	229	222.21	3.35	−2.96	95.25	0.013
Cr	259–319	289	281.29	4.25	−2.67	93.12	4.387

**Table 2:** Contents of heavy metals in sewage sludge and livestock manure (mg·kg^−1^).

Organic Waste	Cu	Zn	Cd	Pb	As	Cr
Sewage sludge	75.96 ± 17.46 a	380.54 ± 209.81 b	0.78 ± 0.16 b	20.16 ± 2.58 b	15.67 ± 5.18 b	180.51 ± 35.45 b
Chicken manure	85.43 ± 38.57 a	395.43 ± 139.96 b	0.31 ± 0.05 a	6.36 ± 4.41 a	2.73 ± 2.26 a	153.66 ± 176.95 b
Swine manure	588.32 ± 315.12 b	933.33 ± 336.28 c	0.33 ± 0.08 a	5.65 ± 3.39 a	6.03 ± 5.17 a	21.86 ± 136.62 a
Cattle manure	41.16 ± 27.27 a	119.52 ± 104.11 a	0.26 ± 0.05 a	13.72 ± 15.28 a	2.59 ± 1.21 a	24.47 ± 34.57 a

Different lower-case letters indicate the results of multiple comparisons between different organic waste.

**Table 3:** Heavy metal limits in sewage sludge and livestock manure for different criteria (mg·kg^−1^).

Organic Waste	Cu	Zn	Cd	Pb	As	Cr
Sewage sludge						
US EPA	1500	2800	39	300	41	1200
European Union Directive 86/278/EEC	1000–1750	2500–4000	20–40	750–1200	-	-
Denmark	1000	4000	0.8	120	25	100
Netherlands	75	300	1.25	100	15	75
Canada	500	2000	20	200	10	1000
GB4284-84						
pH < 6.5	1500	3000	20	1000	75	1000
pH ≥ 6.5	800	2000	5	300	75	600
Fertilizers						
GB8172-87	-	-	3	100	30	300
NY/T 3442-2019	-	-	3	50	15	150
NY525-2012	-	-	3	50	15	150
Quality Control Standards of Imported Organic Fertilizers						
Class I	100	200	0.6	50	5	50
Class II	300	300	1.0	100	10	100

**Table 4:** Results of single-factor pollution index (PI) and Nemerow’s synthetic pollution index (PN) for heavy metals in agricultural use of sewage sludge and livestock manure.

Organic Waste	PI_Cu_	PI_Zn_	PI_Cd_	PI_Pb_	PI_As_	PI_Cr_	PN
Sewage sludge	0.25 a	1.27 b	0.78 b	0.20 b	1.57 b	1.81 b	2.19 b
Chicken manure	0.28 a	1.32 b	0.31 a	0.06 a	0.27 a	1.54 b	2.36 b
Swine manure	1.96 b	3.11 c	0.33 a	0.06 a	0.60 a	0.22 a	3.47 c
Cattle manure	0.14 a	0.40 a	0.26 a	0.14 a	0.26 a	0.24 a	0.52 a

Different lower-case letters indicate the results of multiple comparisons between different organic waste.

**Table 5:** Average daily dose (ADD) of heavy metals in sewage sludge and livestock manure (mg·kg^−1^·day^−1^).

Kinds	Index	Organic Waste	Cu	Zn	Cd	Pb	As	Cr
Adults	ADD_ingest_	Sewage sludge	1.04 × 10^−4^ a	5.21 × 10^−4^ b	4.60 × 10^−7^ b	2.76 × 10^−5^ b	9.20 × 10^−6^ b	2.47 × 10^−4^ b
		Chicken manure	1.17 × 10^−4^ a	5.42 × 10^−4^ b	1.83 × 10^−7^ a	8.72 × 10^−6^ a	1.60 × 10^−6^ a	2.10 × 10^−4^ b
		Swine manure	8.06 × 10^−4^ b	1.28 × 10^−3^ c	1.92 × 10^−7^ a	7.73 × 10^−6^ a	3.54 × 10^−6^ a	2.99 × 10^−5^ a
		Cattle manure	5.64 × 10^−5^ a	1.64 × 10^−4^ a	1.54 × 10^−7^ a	1.88 × 10^−5^ a	1.52 × 10^−6^ a	3.35 × 10^−5^ a
	ADD_inhale_	Sewage sludge	1.53 × 10^−8^ a	7.67 × 10^−8^ b	6.76 × 10^−11^ b	4.06 × 10^−9^ b	1.35 × 10^−9^ b	3.64 × 10^−8^ b
		Chicken manure	1.72 × 10^−8^ a	7.97 × 10^−8^ b	2.69 × 10^−11^ a	1.28 × 10^−9^ a	2.36 × 10^−10^ a	3.10 × 10^−8^ b
		Swine manure	1.19 × 10^−7^ b	1.88 × 10^−7^ c	2.82 × 10^−11^ a	1.14 × 10^−9^ a	5.21 × 10^−10^ a	4.40 × 10^−9^ a
		Cattle manure	8.29 × 10^−9^ a	2.41 × 10^−8^ a	2.27 × 10^−11^ a	2.76 × 10^−9^ a	2.23 × 10^−10^ a	4.93 × 10^−9^ a
	ADD	Sewage sludge	1.04 × 10^−4^ a	5.21 × 10^−4^ b	4.60 × 10^−7^ b	2.76 × 10^−5^ b	9.20 × 10^−6^ b	2.47 × 10^−4^ b
		Chicken manure	1.17 × 10^−4^ a	5.42 × 10^−4^ b	1.83 × 10^−7^ a	8.72 × 10^−6^ a	1.60 × 10^−6^ a	2.11 × 10^−4^ b
		Swine manure	8.06 × 10^−4^ b	1.28 × 10^−3^ c	1.92 × 10^−7^ a	7.73 × 10^−6^ a	3.54 × 10^−6^ a	2.99 × 10^−5^ a
		Cattle manure	5.64 × 10^−5^ a	1.64 × 10^−4^ a	1.54 × 10^−7^ a	1.88 × 10^−5^ a	1.52 × 10^−6^ a	3.35 × 10^−5^ a
Children	ADD_ingest_	Sewage sludge	9.11 × 10^−4^ a	4.56 × 10^−3^ b	8.05 × 10^−7^ b	2.42 × 10^−4^ b	1.61 × 10^−5^ b	2.16 × 10^−3^ b
		Chicken manure	1.02 × 10^−3^ a	4.74 × 10^−3^ b	3.20 × 10^−7^ a	7.63 × 10^−5^ a	2.81 × 10^−6^ a	1.84 × 10^−3^ b
		Swine manure	7.05 × 10^−3^ b	1.12 × 10^−2^ c	3.36 × 10^−7^ a	6.77 × 10^−5^ a	6.20 × 10^−6^ a	2.62 × 10^−4^ a
		Cattle manure	4.93 × 10^−4^ a	1.43 × 10^−3^ a	2.70 × 10^−7^ a	1.64 × 10^−4^ a	2.66 × 10^−6^ a	2.93 × 10^−4^ a
	ADD_inhale_	Sewage sludge	2.54 × 10^−8^ a	1.27 × 10^−7^ b	2.25 × 10^−11^ b	6.75 × 10^−9^ b	4.50 × 10^−10^ b	6.05 × 10^−8^ b
		Chicken manure	2.86 × 10^−8^ a	1.32 × 10^−7^ b	8.94 × 10^−12^ a	2.13 × 10^−9^ a	7.84 × 10^−11^ a	5.15 × 10^−8^ b
		Swine manure	1.97 × 10^−7^ b	3.13 × 10^−7^ c	9.39 × 10^−12^ a	1.89 × 10^−9^ a	1.73 × 10^−10^ a	7.32 × 10^−9^ a
		Cattle manure	1.38 × 10^−8^ a	4.00 × 10^−8^ a	7.54 × 10^−12^ a	4.60 × 10^−9^ a	7.42 × 10^−11^ a	8.20 × 10^−9^ a
	ADD	Sewage sludge	9.11 × 10^−4^ a	4.56 × 10^−3^ b	8.05 × 10^−7^ b	2.42 × 10^−4^ b	1.61 × 10^−5^ b	2.16 × 10^−3^ b
		Chicken manure	1.02 × 10^−3^ a	4.74 × 10^−3^ b	3.20 × 10^−7^ a	7.63 × 10^−5^ a	2.81 × 10^−6^ a	1.84 × 10^−3^ b
		Swine manure	7.05 × 10^−3^ b	1.12 × 10^−2^ c	3.36 × 10^−7^ a	6.77 × 10^−5^ a	6.20 × 10^−6^ a	2.62 × 10^−4^ a
		Cattle manure	4.93 × 10^−4^ a	1.43 × 10^−3^ a	2.70 × 10^−7^ a	1.64 × 10^−4^ a	2.66 × 10^−6^ a	2.93 × 10^−4^ a

Different lower-case letters indicate the results of multiple comparisons between different organic waste.

**Table 6:** Non-carcinogenic risk for adults and children due to environmental exposure to heavy metals in sewage sludge and livestock manure for agricultural use.

Kinds	Index	Organic Waste	Cu	Zn	Pb	Cr
Adults	HQ_ingest_	Sewage sludge	2.60 × 10^−2^ a	1.74 × 10^−3^ b	7.27 × 10^−4^ b	4.95 × 10^−2^ b
		Chicken manure	2.93 × 10^−2^ a	1.81 × 10^−3^ b	2.29 × 10^−4^ a	4.21 × 10^−2^ b
		Swine manure	2.01 × 10^−1^ b	4.26 × 10^−3^ c	2.04 × 10^−4^ a	5.99 × 10^−3^ a
		Cattle manure	1.41 × 10^−2^ a	5.46 × 10^−4^ a	4.95 × 10^−4^ a	6.70 × 10^−3^ a
	HQ_inhale_	Sewage sludge	3.83 × 10^−6^ a	2.56 × 10^−7^ b	1.07 × 10^−7^ b	7.27 × 10^−6^ b
		Chicken manure	4.30 × 10^−6^ a	2.66 × 10^−7^ b	3.37 × 10^−8^ a	6.19 × 10^−6^ b
		Swine manure	2.96 × 10^−5^ b	6.27 × 10^−7^ c	2.99 × 10^−8^ a	8.81 × 10^−7^ a
		Cattle manure	2.07 × 10^−6^ a	8.03 × 10^−8^ a	7.27 × 10^−8^ a	9.86 × 10^−7^ a
	HQ	Sewage sludge	2.60 × 10^−2^ a	1.74 × 10^−3^ b	7.27 × 10^−4^ b	4.95 × 10^−2^ b
		Chicken manure	2.93 × 10^−2^ a	1.81 × 10^−3^ b	2.29 × 10^−4^ a	4.21 × 10^−2^ b
		Swine manure	2.02 × 10^−1^ b	4.26 × 10^−3^ c	2.04 × 10^−4^ a	5.99 × 10^−3^ a
		Cattle manure	1.41 × 10^−2^ a	5.46 × 10^−4^ a	4.95 × 10^−4^ a	6.71 × 10^−3^ a
	HI	Sewage sludge	7.79 × 10^−2^ a			
		Chicken manure	7.34 × 10^−2^ a			
		Swine manure	2.12 × 10^−1^ b			
		Cattle manure	2.18 × 10^−2^ a			
Children	HQ_ingest_	Sewage sludge	2.28 × 10^−1^ a	1.52 × 10^−2^ b	6.36 × 10^−3^ b	4.33 × 10^−1^ b
		Chicken manure	2.56 × 10^−1^ a	1.58 × 10^−2^ b	2.01 × 10^−3^ a	3.68 × 10^−1^ b
		Swine manure	1.76 b	3.73 × 10^−2^ c	1.78 × 10^−3^ a	5.24 × 10^−2^ a
		Cattle manure	1.23 × 10^−1^ a	4.78 × 10^−3^ a	4.33 × 10^−3^ a	5.87 × 10^−2^ a
	HQ_inhale_	Sewage sludge	6.36 × 10^−6^ a	4.25 × 10^−7^ b	1.78 × 10^−7^ b	1.21 × 10^−5^ b
		Chicken manure	7.15 × 10^−6^ a	4.41 × 10^−7^ b	5.61 × 10^−8^ a	1.03 × 10^−5^ b
		Swine manure	4.93 × 10^−5^ b	1.04 × 10^−6^ c	4.98 × 10^−8^ a	1.46 × 10^−6^ a
		Cattle manure	3.45 × 10^−6^ a	1.33 × 10^−7^ a	1.21 × 10^−7^ a	1.64 × 10^−6^ a
	HQ	Sewage sludge	2.28 × 10^−1^ a	1.52 × 10^−2^ b	6.36 × 10^−3^ b	4.33 × 10^−1^ b
		Chicken manure	2.56 × 10^−1^ a	1.58 × 10^−2^ b	2.01 × 10^−3^ a	3.68 × 10^−1^ b
		Swine manure	1.76 b	3.73 × 10^−2^ c	1.78 × 10^−3^ a	5.24 × 10^−2^ a
		Cattle manure	1.23 × 10^−1^ a	4.78 × 10^−3^ a	4.33 × 10^−3^ a	5.87 × 10^−2^ a
	HI	Sewage sludge	6.82 × 10^−1^ a			
		Chicken manure	6.42 × 10^−1^ a			
		Swine manure	1.85 b			
		Cattle manure	1.91 × 10^−1^ a			

Different lower-case letters indicate the results of multiple comparisons between different organic waste.

**Table 7:** Carcinogenic risk for adults and children due to environmental exposure to heavy metals in sewage sludge and livestock manure for agricultural use.

Kinds	Organic Waste	As			Cd			RISK
		Risk_ingest_	Risk_inhale_	Risk	Risk_ingest_	Risk_inhale_	Risk	
Adults	Sewage sludge	1.38 × 10^−5^ b	2.03 × 10^−9^ b	1.38 × 10^−5^ b	2.81 × 10^−6^ b	4.13 × 10^−10^ b	2.81 × 10^−6^ b	1.66 × 10^−5^ b
	Chicken manure	2.41 × 10^−6^ a	3.54 × 10^−10^ a	2.41 × 10^−6^ a	1.12 × 10^−6^ a	1.64 × 10^−10^ a	1.12 × 10^−6^ a	3.52 × 10^−6^ a
	Swine manure	5.31 × 10^−6^ a	7.81 × 10^−10^ a	5.31 × 10^−6^ a	1.17 × 10^−6^ a	1.72 × 10^−10^ a	1.17 × 10^−6^ a	6.48 × 10^−6^ a
	Cattle manure	2.28 × 10^−6^ a	3.35 × 10^−10^ a	2.28 × 10^−6^ a	9.41 × 10^−7^ a	1.38 × 10^−10^ a	9.41 × 10^−7^ a	3.22 × 10^−6^ a
Children	Sewage sludge	2.41 × 10^−5^ b	6.75 × 10^−10^ b	2.41 × 10^−5^ b	4.91 × 10^−6^ b	1.37 × 10^−10^ b	4.91 × 10^−6^ b	2.91 × 10^−5^ b
	Chicken manure	4.21 × 10^−6^ a	1.18 × 10^−10^ a	4.21 × 10^−6^ a	1.95 × 10^−6^ a	5.45 × 10^−11^ a	1.95 × 10^−6^ a	6.16 × 10^−6^ a
	Swine manure	9.30 × 10^−6^ a	2.60 × 10^−10^ a	9.30 × 10^−6^ a	2.05 × 10^−6^ a	5.73 × 10^−11^ a	2.05 × 10^−6^ a	1.13 × 10^−5^ a
	Cattle manure	3.99 × 10^−6^ a	1.11 × 10^−10^ a	3.99 × 10^−6^ a	1.65 × 10^−6^ a	4.60 × 10^−11^ a	1.65 × 10^−6^ a	5.63 × 10^−6^ a

Different lower-case letters indicate the results of multiple comparisons between different organic waste.

## Data Availability

The data presented in this study are available in the article and in the Supplementary Materials.

## Supplementary Materials

The following are available online at https://www.mdpi.com/article/10.3390/toxics9070145/s1, Table S1: Analytical accuracy, precision, recovery and method detection limit; Table S2: Contents of heavy metals in sewage sludge and livestock manure (mg·kg^−1^); Table S3: Heavy metal limits in sewage sludge and livestock manure for different criteria (mg·kg^−1^); Table S4: Results of single-factor pollution index (PI) and Nemerow’s synthetic pollution index (PN) for heavy metals in agricultural use of sewage sludge and livestock manure; Table S5: Average daily dose (ADD) of heavy metals in sewage sludge and livestock manure (mg·kg^−1^·day^−1^); Table S6: Non-carcinogenic risk for adults and children due to environmental exposure to heavy metals in sewage sludge and livestock manure for agricultural use; Table S7: Carcinogenic risk for adults and children due to environmental exposure to heavy metals in sewage sludge and livestock manure for agricultural use.
