# Supplementary material for: Comparison of the Potential Ecological and Human Health Risks of Heavy Metals from Sewage Sludge and Livestock Manure for Agricultural Use
Source: Toxics. 2021 Jun 24;9(7):145. doi: 10.3390/toxics9070145 (PMC8309916; doi:10.3390/toxics9070145)
Supplement: Supplementary file 1 [file toxics-09-00145-s001.zip › toxics-1218579-supplementary.pdf]

toxics

MDPI

Article

Supplementary Materials: Comparison of the Potential Ecological and Human Health Risks of Heavy Metals from Sewage Sludge and Livestock Manure for Agricultural Use

Baoling Duan and Qiang Feng

Table S1. Analytical accuracy, precision, recovery and method detection limit.

Heavy Metal	Confidence Interval (mg/kg)	Certified Value (mg/kg)	Measured Value (mg/kg)	Accuracy (%)	Precision (%)	Recovery (%)	Method Detection Limit (mg/kg)
Cu	433-531	482	470.96	3.68	-2.29	94.61	0.907
Zn	1060-1420	1240	1229.59	5.02	-0.84	94.51	0.984
Cd	56.9-64.2	60	63.01	1.43	5.01	105.92	0.0063
Pb	143-165	154	147.79	3.38	-4.03	92.77	0.318
As	202-256	229	222.21	3.35	-2.96	95.25	0.013
Cr	259-319	289	281.29	4.25	-2.67	93.12	4.387

Table S2. Contents of heavy metals in sewage sludge and livestock manure (mg·kg^{-1}).

Organic waste	Cu	Zn	Cd	Pb	As	Cr
Sewage sludge	75.96±17.46 a	380.54±209.81 b	0.78±0.16 b	20.16±2.58 b	15.67±5.18 b	180.51±35.45 b
Chicken manure	85.43±38.57 a	395.43±139.96 b	0.31±0.05 a	6.36±4.41 a	2.73±2.26 a	153.66±176.95 b
Swine manure	588.32±315.12 b	933.33±336.28 c	0.33±0.08 a	5.65±3.39 a	6.03±5.17 a	21.86±136.62 a
Cattle manure	41.16±27.27 a	119.52±104.11 a	0.26±0.05 a	13.72±15.28 a	2.59±1.21 a	24.47±34.57 a

Different lower-case letters indicate the result of multiple comparisons between different organic waste.

Table S3. Heavy metal limits in sewage sludge and livestock manure for different criteria (mg·kg^{-1}).

Organic waste	Cu	Zn	Cd	Pb	As	Cr
Sewage sludge						
USEPA	1500	2800	39	300	41	1200
European Union	1000–1750	2500–4000	20–40	750–1200	-	-
Canada	500	2000	20	200	10	1000
GB4284-84						
pH < 6.5	1500	3000	20	1000	75	1000
pH ≥ 6.5	800	2000	5	300	75	600
Fertilizers						
GB8172-87	-	-	3	100	30	300
NY/T 3442-2019	-	-	3	50	15	150
NY525-2012	-	-	3	50	15	150
Quality Control Standards of Imported Organic Fertilizers						
First standard	100	200	0.6	50	5	50
Secondary standard	300	300	1.0	100	10	100

Table S4. Results of single factor pollution index (PI) and Nemerow's synthetic pollution index (PN) for heavy metals in agricultural use of sewage sludge and livestock manure.

Organic waste	PI_{Cu}	PI_{Zn}	PI_{Cd}	PI_{Pb}	PI_{As}	PI_{Cr}	PN
Sewage sludge	0.25 a	1.27 b	0.78 b	0.20 b	1.57 b	1.81 b	2.19 b
Chicken manure	0.28 a	1.32 b	0.31 a	0.06 a	0.27 a	1.54 b	2.36 b
Swine manure	1.96 b	3.11 c	0.33 a	0.06 a	0.60 a	0.22 a	3.47 c
Cattle manure	0.14 a	0.40 a	0.26 a	0.14 a	0.26 a	0.24 a	0.52 a

Different lower-case letters indicate the result of multiple comparisons between different organic waste.

Table S5. Average daily dose (ADD) of heavy metals in sewage sludge and livestock manure ($mg \cdot kg^{-1} \cdot day^{-1}$).

Kinds	Index	Organic waste	Cu	Zn	Cd	Pb	As	Cr
Adults	ADD_{ingest}	Sewage sludge	1.04E-04 a	5.21E-04 b	4.60E-07 b	2.76E-05 b	9.20E-06 b	2.47E-04 b
		Chicken manure	1.17E-04 a	5.42E-04 b	1.83E-07 a	8.72E-06 a	1.60E-06 a	2.10E-04 b
		Swine manure	8.06E-04 b	1.28E-03 c	1.92E-07 a	7.73E-06 a	3.54E-06 a	2.99E-05 a
		Cattle manure	5.64E-05 a	1.64E-04 a	1.54E-07 a	1.88E-05 a	1.52E-06 a	3.35E-05 a
	ADD_{inhale}	Sewage sludge	1.53E-08 a	7.67E-08 b	6.76E-11 b	4.06E-09 b	1.35E-09 b	3.64E-08 b
		Chicken manure	1.72E-08 a	7.97E-08 b	2.69E-11 a	1.28E-09 a	2.36E-10 a	3.10E-08 b
		Swine manure	1.19E-07 b	1.88E-07 c	2.82E-11 a	1.14E-09 a	5.21E-10 a	4.40E-09 a
		Cattle manure	8.29E-09 a	2.41E-08 a	2.27E-11 a	2.76E-09 a	2.23E-10 a	4.93E-09 a
	ADD	Sewage sludge	1.04E-04 a	5.21E-04 b	4.60E-07 b	2.76E-05 b	9.20E-06 b	2.47E-04 b
		Chicken manure	1.17E-04 a	5.42E-04 b	1.83E-07 a	8.72E-06 a	1.60E-06 a	2.11E-04 b
		Swine manure	8.06E-04 b	1.28E-03 c	1.92E-07 a	7.73E-06 a	3.54E-06 a	2.99E-05 a
		Cattle manure	5.64E-05 a	1.64E-04 a	1.54E-07 a	1.88E-05 a	1.52E-06 a	3.35E-05 a
Children	ADD_{ingest}	Sewage sludge	9.11E-04 a	4.56E-03 b	8.05E-07 b	2.42E-04 b	1.61E-05 b	2.16E-03 b
		Chicken manure	1.02E-03 a	4.74E-03 b	3.20E-07 a	7.63E-05 a	2.81E-06 a	1.84E-03 b
		Swine manure	7.05E-03 b	1.12E-02 c	3.36E-07 a	6.77E-05 a	6.20E-06 a	2.62E-04 a
		Cattle manure	4.93E-04 a	1.43E-03 a	2.70E-07 a	1.64E-04 a	2.66E-06 a	2.93E-04 a
	ADD_{inhale}	Sewage sludge	2.54E-08 a	1.27E-07 b	2.25E-11 b	6.75E-09 b	4.50E-10 b	6.05E-08 b
		Chicken manure	2.86E-08 a	1.32E-07 b	8.94E-12 a	2.13E-09 a	7.84E-11 a	5.15E-08 b
		Swine manure	1.97E-07 b	3.13E-07 c	9.39E-12 a	1.89E-09 a	1.73E-10 a	7.32E-09 a
		Cattle manure	1.38E-08 a	4.00E-08 a	7.54E-12 a	4.60E-09 a	7.42E-11 a	8.20E-09 a
	ADD	Sewage sludge	9.11E-04 a	4.56E-03 b	8.05E-07 b	2.42E-04 b	1.61E-05 b	2.16E-03 b
		Chicken manure	1.02E-03 a	4.74E-03 b	3.20E-07 a	7.63E-05 a	2.81E-06 a	1.84E-03 b
		Swine manure	7.05E-03 b	1.12E-02 c	3.36E-07 a	6.77E-05 a	6.20E-06 a	2.62E-04 a
		Cattle manure	4.93E-04 a	1.43E-03 a	2.70E-07 a	1.64E-04 a	2.66E-06 a	2.93E-04 a

Different lower-case letters indicate the result of multiple comparisons between different organic waste.

Table S6. Non-carcinogenic risk for adults and children due to environmental exposure to heavy metals in sewage sludge and livestock manure for agricultural use.

Kinds	Index	Organic waste	Cu	Zn	Pb	Cr
Adults	HQ_{ingest}	Sewage sludge	2.60E-02 a	1.74E-03 b	7.27E-04 b	4.95E-02 b
		Chicken manure	2.93E-02 a	1.81E-03 b	2.29E-04 a	4.21E-02 b
		Swine manure	2.01E-01 b	4.26E-03 c	2.04E-04 a	5.99E-03 a
		Cattle manure	1.41E-02 a	5.46E-04 a	4.95E-04 a	6.70E-03 a
	HQ_{inhale}	Sewage sludge	3.83E-06 a	2.56E-07 b	1.07E-07 b	7.27E-06 b
		Chicken manure	4.30E-06 a	2.66E-07 b	3.37E-08 a	6.19E-06 b
		Swine manure	2.96E-05 b	6.27E-07 c	2.99E-08 a	8.81E-07 a
		Cattle manure	2.07E-06 a	8.03E-08 a	7.27E-08 a	9.86E-07 a

	HQ	Sewage sludge	2.60E-02 a	1.74E-03 b	7.27E-04 b	4.95E-02 b
		Chicken manure	2.93E-02 a	1.81E-03 b	2.29E-04 a	4.21E-02 b
		Swine manure	2.02E-01 b	4.26E-03 c	2.04E-04 a	5.99E-03 a
		Cattle manure	1.41E-02 a	5.46E-04 a	4.95E-04 a	6.71E-03 a
	HI	Sewage sludge	7.79E-02 a			
		Chicken manure	7.34E-02 a			
		Swine manure	2.12E-01 b			
		Cattle manure	2.18E-02 a			
Children	HQ_{ingest}	Sewage sludge	2.28E-01 a	1.52E-02 b	6.36E-03 b	4.33E-01 b
		Chicken manure	2.56E-01 a	1.58E-02 b	2.01E-03 a	3.68E-01 b
		Swine manure	1.76E+00 b	3.73E-02 c	1.78E-03 a	5.24E-02 a
		Cattle manure	1.23E-01 a	4.78E-03 a	4.33E-03 a	5.87E-02 a
	HQ_{inhale}	Sewage sludge	6.36E-06 a	4.25E-07 b	1.78E-07 b	1.21E-05 b
		Chicken manure	7.15E-06 a	4.41E-07 b	5.61E-08 a	1.03E-05 b
		Swine manure	4.93E-05 b	1.04E-06 c	4.98E-08 a	1.46E-06 a
		Cattle manure	3.45E-06 a	1.33E-07 a	1.21E-07 a	1.64E-06 a
	HQ	Sewage sludge	2.28E-01 a	1.52E-02 b	6.36E-03 b	4.33E-01 b
		Chicken manure	2.56E-01 a	1.58E-02 b	2.01E-03 a	3.68E-01 b
		Swine manure	1.76E+00 b	3.73E-02 c	1.78E-03 a	5.24E-02 a
		Cattle manure	1.23E-01 a	4.78E-03 a	4.33E-03 a	5.87E-02 a
	HI	Sewage sludge	6.82E-01 a			
		Chicken manure	6.42E-01 a			
		Swine manure	1.85E+00 b			
		Cattle manure	1.91E-01 a			

Different lower-case letters indicate the result of multiple comparisons between different organic waste.

Table 7. Carcinogenic risk for adults and children due to environmental exposure to heavy metals in sewage sludge and livestock manure for agricultural use.

Kinds	Organic Waste	As			Cd			RISK
		$Risk_{ingest}$	$Risk_{inhale}$	Risk	$Risk_{ingest}$	$Risk_{inhale}$	Risk	
Adults	Sewage sludge	1.38E-05 b	2.03E-09 b	1.38E-05 b	2.81E-06 b	4.13E-10 b	2.81E-06 b	1.66E-05 b
	Chicken manure	2.41E-06 a	3.54E-10 a	2.41E-06 a	1.12E-06 a	1.64E-10 a	1.12E-06 a	3.52E-06 a
	Swine manure	5.31E-06 a	7.81E-10 a	5.31E-06 a	1.17E-06 a	1.72E-10 a	1.17E-06 a	6.48E-06 a
	Cattle manure	2.28E-06 a	3.35E-10 a	2.28E-06 a	9.41E-07 a	1.38E-10 a	9.41E-07 a	3.22E-06 a
Children	Sewage sludge	2.41E-05 b	6.75E-10 b	2.41E-05 b	4.91E-06 b	1.37E-10 b	4.91E-06 b	2.91E-05 b
	Chicken manure	4.21E-06 a	1.18E-10 a	4.21E-06 a	1.95E-06 a	5.45E-11 a	1.95E-06 a	6.16E-06 a
	Swine manure	9.30E-06 a	2.60E-10 a	9.30E-06 a	2.05E-06 a	5.73E-11 a	2.05E-06 a	1.13E-05 a
	Cattle manure	3.99E-06 a	1.11E-10 a	3.99E-06 a	1.65E-06 a	4.60E-11 a	1.65E-06 a	5.63E-06 a

Different lower-case letters indicate the result of multiple comparisons between different organic waste.
